# From sixty-four percent hyperparasitemia to recovery: Managing severe falciparum malaria and delayed hemolytic anemia

**DOI:** 10.1016/j.ijregi.2025.100644

**Published:** 2025-04-09

**Authors:** Fiona Murphy, Tanna Tan, Josephine Hebert, Cora Mc Nally

**Affiliations:** 1 Infectious Diseases, Beaumont Hospital, Dublin, Ireland; 2 Haematology, Beaumont Hospital, Dublin, Ireland

**Keywords:** Hyperparasitemia, Malaria, Artesunate, Post-artesunate delayed hemolysis (PADH)

## Abstract

**Objectives:**

Severe *Plasmodium falciparum* malaria with hyperparasitemia is a life-threatening condition requiring urgent intervention, particularly in non-immune individuals. Artesunate has revolutionized its management, though complications such as post-artesunate delayed hemolysis (PADH) necessitate ongoing vigilance.

**Methods:**

We report a case of a 26-year-old sailor with a history of recent travel to Nigeria who presented to an Irish hospital with severe falciparum malaria and hyperparasitemia of 64.35%. Initial management included intravenous artesunate, continuous renal replacement therapy, and hemodynamic support, leading to rapid parasite clearance and recovery from multi-organ dysfunction. However, 1 week post-discharge, the patient developed PADH, manifesting as severe anemia requiring blood transfusion.

**Results:**

This case underscores the importance of prompt diagnosis, rapid parasite clearance with artesunate, and multidisciplinary supportive care in severe malaria. Furthermore, the occurrence of PADH highlights the need for structured post-treatment follow-up, particularly in non-endemic regions where clinicians may be unfamiliar with such complications.

**Conclusions:**

Severe falciparum malaria requires urgent and aggressive management. While artesunate is life-saving, its delayed complications necessitate systematic monitoring. This case highlights the importance of integrating acute care with vigilant follow-up to optimize outcomes in severe malaria, especially in non-immune individuals with hyperparasitemia.

## Highlights

• Hyperparasitemia (64%) in a non-immune traveler can cause multiorgan dysfunction.
• Post-artesunate delayed hemolysis (PADH) emerged as a delayed complication.
• Intravenous artesunate cleared parasites rapidly but required vigilant PADH management.
• Clinicians in non-endemic areas must recognize and manage PADH early.
• Post-treatment follow-up is crucial for optimizing outcomes in severe malaria.

## Introduction

This case report presents a severe malaria case from a traveler to Nigeria. In 2023, the World Health Organization (WHO) reported that Nigeria was among the top five countries with the heaviest malaria burden, contributing 26% of global malaria cases [1]. Severe falciparum malaria is a medical emergency, particularly in non-immune individuals, and hyperparasitemia (parasitemia >5%) significantly increases the risk of mortality [2]. Artesunate has revolutionized the management of severe malaria, offering rapid parasite clearance and improved survival compared to quinine [3]. Post-artesunate delayed hemolysis (PADH) is a delayed complication of severe malaria treatment with intravenous artesunate, occurring 1-3 weeks post-treatment. It is marked by worsening anemia and laboratory signs of hemolysis, including elevated lactate dehydrogenase (LDH), reticulocytosis, and reduced haptoglobin [4]. Though its mechanism is not fully understood, PADH likely involves immune-mediated or oxidative damage to red blood cells [5]. This report presents a case of severe falciparum malaria with hyperparasitemia of 64.35%, highlighting the importance of rapid intervention and long-term monitoring.

## Case presentation

A 26-year-old sailor from Siberia presented to an Irish hospital with a 5-day history of fever, abdominal pain, nausea, and malaise. He had recently traveled to Nigeria without taking malaria prophylaxis. On admission, he was hypotensive (blood pressure 80/50 mmHg), tachycardic (heart rate 118 bpm), and oliguric. Clinical examination revealed fluid overload with elevated jugular venous pressure, bibasal crackles, and 3+ pitting edema to the knees. His heart sounds were normal, and his abdomen was soft and non-tender.

Laboratory investigations showed severe thrombocytopenia and an acute kidney injury (AKI) (Table 1).

**Table 1:** Laboratory investigations on admission.

Laboratory test	Value	Normal range
Hemoglobin	5.2 g/dl	14-17.5 g/dl
Lactate dehydrogenase	1786 U/l	140-280 U/l
Reticulocytes	298 × 10⁹/l	0.5-1.5 × 10⁹/l
Bilirubin	108 µmol/l	3 to 17 µmol/l
Haptoglobin	22 mg/dl	41-165 mg/dl
Malaria film	Negative	

**Figure 1:**
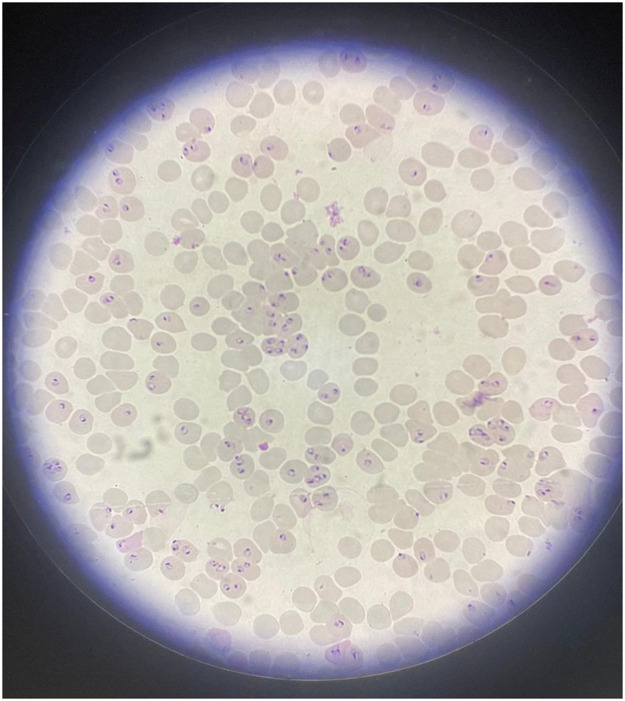
Blood film.

A rapid malaria test confirmed *Plasmodium falciparum*. A peripheral blood film revealed a hyperparasitemia of 64.35% (Figure 1).

The patient was admitted to the intensive care unit and commenced on noradrenaline for hemodynamic support. Continuous renal replacement therapy (CRRT) was initiated to manage AKI and fluid overload. Artesunate is stocked as an emergency medicine in Irish hospitals. Intravenous artesunate was administered within 6 hours of admission at 2.4 mg/kg at 0, 12, and 24 hours, leading to a significant reduction in parasitemia to 7.5% within 24 hours and complete clearance by 48 hours. After 72 hours of treatment, he was transitioned to oral artemether-lumefantrine and completed a treatment course, as per international guidelines [6,7]. Intravenous artesunate is widely recognized as the gold standard treatment for severe malaria and has fundamentally transformed its management [7].

The patient showed marked improvement, with resolution of thrombocytopenia and coagulopathy. He was weaned off vasopressors, and CRRT was discontinued as urine output normalized. He was discharged 5 days after artesunate, his hemoglobin on discharge was 13.8 g/dl. However, 10 days after starting artesunate, he re-presented with severe anemia and was jaundiced (Table 2). Laboratory investigations revealed elevated lactate dehydrogenase, reticulocytosis, and a negative antibody screen, confirming PADH. He required a weeklong admission and transfusion of four units of packed red blood cells throughout the course of his stay before his hemoglobin stabilized prior to discharge.

**Table 2:** Laboratory investigations 10 days after commencing intravenous artesunate.

Laboratory test	Admission value	Normal range
Hemoglobin	14.6 g/dl	14-17.5 g/dl
Platelets	14 × 10⁹/l	150-450 × 10⁹/l
Urea	20.6 mmol/l	2.5-7.8 mmol/l
Creatinine	361 µmol/l	44-133 µmol/l
Lactate	13 mmol/l	<2 mmol/l
INR	2.42	<1.1
Alanine aminotransferase	114 U/l	7-56 U/l
Aspartate aminotransferase	97 U/l	10-40 U/l

## Discussion

Malaria is a vector-borne infectious parasite that is transmitted through mosquitos [8]. This case highlights the significant challenges associated with managing severe falciparum malaria with hyperparasitemia, particularly in a non-immune individual. Hyperparasitemia is defined differently by various groups. WHO in their most recent guidelines states, “In low-transmission settings, mortality begins to increase when the parasite density exceeds 100,000/µl (∼2% parasitemia)” [1]. Some non-endemic countries recommend IV artesunate treatment at a parasitemia of 2% or greater, although the Centre for Disease Control and Prevention (CDC) uses 5% if there are no other signs/symptoms of severe disease as criteria for treating with intravenous (IV) artesunate [9].

Hyperparasitemia represents a critical clinical condition due to its association with multiorgan dysfunction and a markedly increased risk of mortality [10]. The parasitemia of 64.35% observed in this patient underscores the life-threatening nature of severe malaria and demonstrates the need for urgent and aggressive management to improve outcomes. In non-immune individuals, such as travelers from malaria-free regions, the absence of pre-existing immunity to *P. falciparum* places them at an even higher risk of developing severe complications [11]. This case reinforces the necessity of prompt recognition and treatment to mitigate the substantial mortality risk posed by severe malaria.

Intravenous artesunate is widely recognized as the gold standard treatment for severe malaria and has fundamentally transformed its management [7]. Artesunate's superior efficacy and safety compared to quinine have been demonstrated in large-scale clinical trials, making it the preferred agent for severe cases [12]. Artesunate rapidly kills ring-stage parasites, which are then taken out of the red cells by the spleen; these infected erythrocytes are then returned to circulation but with a shortened life span, resulting in the observed hemolysis [4]. In this patient, artesunate facilitated a dramatic reduction in parasitemia to 7.5% within 24 hours and complete clearance by 48 hours. Such outcomes highlight the drug's efficacy even in extreme cases of hyperparasitemia. The transition to oral artemether-lumefantrine following clearance of peripheral parasitemia aligns with current WHO guidelines, which recommend combination therapy to sustain therapeutic efficacy and minimize the risk of recrudescence [1].

While artesunate is a life-saving treatment, its use is associated with PADH, a complication that requires careful post-treatment monitoring [4].

PADH is most commonly observed in patients with high parasite burdens and is thought to result from the delayed splenic clearance of non-viable, parasitized erythrocytes that persist in circulation [4]. This phenomenon often manifests 1-3 weeks after treatment initiation and can lead to significant anemia. A study reported that the incidence of PADH was doubled in European patients, compared with those of African origin [13].

In this case, the patient's development of severe anemia (hemoglobin 5.2 g/dl) 10 days after IV artesunate, along with elevated lactate dehydrogenase and reticulocytosis, was consistent with PADH. While PADH is typically self-limiting, the severity of anemia in this patient necessitated transfusions of packed red blood cells, emphasizing the importance of post-treatment vigilance. Recent findings from the CDC have documented similar presentations in non-endemic regions, including the United States, reinforcing the importance of systematic follow-up to detect and manage PADH effectively [14]. These observations underline the need for clinicians in non-endemic regions to maintain a high index of suspicion for delayed complications following artesunate treatment.

The successful management of this case also highlights the critical role of supportive care in treating severe malaria with hyperparasitemia. This patient exhibited multiorgan dysfunction, including AKI, thrombocytopenia, and coagulopathy, all of which are common complications in severe malaria. The pathophysiology of these complications often involves microvascular obstruction and systemic inflammation driven by the sequestration of infected erythrocytes. In this context, CRRT was instrumental in managing oliguric AKI and fluid overload, while platelet transfusions and vasopressors provided additional support during the acute phase of the illness. The resolution of organ dysfunction following parasite clearance underscores the importance of integrating targeted antimalarial therapy with multidisciplinary supportive measures. This case demonstrates that a coordinated approach, combining rapid parasite clearance with aggressive supportive care, is essential for optimizing patient outcomes in severe malaria.

The implications of this case extend beyond the individual patient and have broader relevance for the management of malaria in non-endemic regions. The rarity of malaria in such settings often leads to diagnostic delays and unfamiliarity with treatment protocols, increasing the risk of severe complications. This case underscores the importance of a thorough travel history in the evaluation of febrile illnesses, as prompt recognition of imported malaria can significantly alter clinical outcomes. Additionally, it highlights the need for enhanced awareness among clinicians regarding post-treatment complications such as PADH, which, while well-documented, remains underreported in non-endemic regions. The CDC's recent data on PADH cases in the United States emphasize the critical importance of structured post-treatment follow-up to monitor for delayed hemolysis and anemia. If PADH occurs, patients need routine lab work done for 4 weeks to monitor hemoglobin, reticulocyte count, haptoglobin, lactate dehydrogenase, and total bilirubin [4].

## Conclusion

In conclusion, this case exemplifies the transformative role of artesunate in managing severe hyperparasitemia and underscores the necessity of early intervention and multidisciplinary supportive care in addressing the complications of severe malaria. It also emphasizes the need for post-treatment vigilance, particularly in non-immune individuals with high parasite burdens, to detect and manage delayed sequelae such as PADH. Through the integration of acute management and systematic follow-up, optimal outcomes can be achieved even in the most severe cases of falciparum malaria.

## Declarations of competing interest

The authors have no competing interests to declare.
